# Reliability and Effectiveness of the Japanese Version of the Mobilization Quantification Score

**DOI:** 10.7759/cureus.43440

**Published:** 2023-08-13

**Authors:** Shinichi Watanabe, Kota Yamauchi, Daisetsu Yasumura, Keisuke Suzuki, Takayasu Koike, Hajime Katsukawa, Yasunari Morita, Flora T Scheffenbichler, Stefan J Schaller, Matthias Eikermann

**Affiliations:** 1 Department of Physical Therapy, Faculty of Rehabilitation, Gifu University of Medical Science, Gifu, JPN; 2 Department of Rehabilitation Medicine, Nagoya Medical Center, National Hospital Organization, Nagoya, JPN; 3 Department of Rehabilitation Medicine, Steel Memorial Yawata Hospital, Kitakyushu, JPN; 4 Department of Rehabilitation Medicine, Naha City Hospital, Okinawa, JPN; 5 Department of Physical Therapy, Gifu University of Health Science, Gifu, JPN; 6 Department of Physical Therapy, Faculty of Rehabilitation, Gifu University of Health Science, Gifu, JPN; 7 Physical Medicine and Rehabilitation, Japanese Society for Early Mobilization, Tokyo, JPN; 8 Department of Emergency Medicine, Nagoya Medical Center, Nagoya, JPN; 9 Department of Anesthesiology and Intensive Care Medicine, Ulm University, Ulm, DEU; 10 Department of Anesthesiology and Intensive Care, Technical University of Munich, Munich, DEU; 11 Department of Anesthesiology and Operative Intensive Care, Charité – Universitätsmedizin Berlin, Corporate Member of Freie Universität Berlin and Humboldt-Universität zu Berlin, Berlin, DEU; 12 Department of Anesthesiology, Montefiore Medical Center, Albert Einstein College of Medicine, New York, USA

**Keywords:** reliability, rehabilitation, mechanical ventilation, activity of daily living (adl), early mobilization

## Abstract

Background

The mobilization quantification score (MQS) provides an opportunity to quantify the duration and intensity of mobilization therapy in the intensive care unit (ICU) and predict functional outcomes in ICU patients after surgery and stroke. MQS is a numerical measurement of early mobilization dose in the ICU, and its relationship with activities of daily living (ADL) dependence has been shown. We created and validated the Japanese version of the MQS using the endpoint ADL in a mixed population of patients in the ICU.

Materials and methods

In this prospective study, consecutive patients who were admitted to one of three ICUs of a tertiary care hospital in Japan, aged ≥18 years, and who received mechanical ventilation for >48 hours were enrolled. The Japanese version of the MQS was applied twice daily by an ICU physiotherapist and data recorded for analysis. The primary outcome was ADL dependence at hospital discharge, defined as a Barthel index (BI) of <70 or in-hospital death. The reliability among assessors was verified by calculating the interclass correlation coefficient (ICC) (2.1) for the average daily MQS. We performed a multiple logistic regression analysis to examine and identify a binary cutoff point for high-/low-dose rehabilitation.

Results

Of the 340 target patients, eight were aged <18 years, 109 had neurological complications, 11 had a BI <70 before admission, 79 had a lack of communication skills, 16 were terminally ill, eight did not complete the assessment during their ICU stay, 18 died in the ICU, and 53 denied consent. After 302 patients were excluded, 38 were included in the study. Six assessors, two at each hospital, measured the MQS in 38 patients. The ICC (2.1) for the MQS mean value was 0.98 (0.96-0.99) during the ICU stay. Logistic regression analysis using the mean MQS on admission to ICUs as an explanatory variable showed a significant association between increased MQS and decreased ADL dependence at discharge (odds ratio (OR): 0.76, confidence interval (CI): 0.61-0.96, adjusted p = 0.009). Logistic regression analysis using a high MQS on admission to ICUs as an explanatory variable showed a significant association between increased MQS and decreased ADL dependence at hospital discharge (OR: 0.14, CI: 0.03-0.66, adjusted p = 0.013).

Conclusions

We present a validated version of the Japanese MQS with a high inter-rater reliability that predicts ADL dependence at hospital discharge. The instrument can be used in future clinical trials in the ICU to control for the mobilization level in the ICU. The increased utilization of mobilization acutely in the ICU setting as quantified by the MQS may improve patient outcomes.

## Introduction

Post-intensive care syndrome (PICS), a general term for motor and cognitive dysfunction that becomes apparent after the acute phase of intensive care, has attracted attention in recent years [1]. PICS causes physical disability, cognitive dysfunction, and psychiatric disorders that develop or worsen in patients after recovery from severe acute conditions, such as severe sepsis and acute respiratory distress syndrome [2,3]. Early rehabilitation is one of the preventive methods; however, further investigation into the specific clinical strength and activity time is needed [4-6]. In addition, in Japan, variations in early rehabilitation programs based on the hospital environment and the experience of the therapist still exist, and many hospitals have not created evidence-based protocols [7].

Physical activity (PA) in patients with respiratory failure is a significant prognostic factor for mortality due to severe exacerbation [8,9]. In particular, PA in patients with severe respiratory failure tends to decrease due to disease management [10]. In addition, PA is often difficult to improve because of severe shortness of breath and impaired exercise tolerance. However, some patients are unable to take the step of mobilization to standing and walking while in the intensive care unit (ICU), and even minimal exercise activity can reduce weakness and wasting [11].

A previous study by Scheffenbichler et al. created a new tool for assessing mobilization doses based on the level and duration of rehabilitation (mobilization quantification score, MQS) [12]. The MQS was developed based on expert opinions, and its reproducibility has been verified at multiple health centers. In addition, the MQS is a numerical measure of the dose of early mobilization in the ICU and has been shown to predict mortality and adverse discharge [12,13].

There are several benefits associated with using the MQS to assess PA for the early rehabilitation of patients in the ICU. Owing to the peculiarities of the ICU environment, it is difficult to evaluate the amount of PA using a PA meter or metabolic measuring instrument, and it is important to develop a simple evaluation scale, such as the MQS [12,13]. The MQS has excellent concurrency, inter-racial consensus, and no caps and provides consistent results among professionals. The MQS is available in English; however, Japanese versions are not currently available. Therefore, this study aimed to develop a Japanese version of the MQS and confirm its reliability and effectiveness. This study evaluated whether the MQS could predict activity of daily living (ADL) dependency at hospital discharge in patients with mechanical ventilation in mixed ICUs in Japan. We also evaluated the inter-rater reliability of the Japanese version of the MQS.

## Materials and methods

Methods

The reliability and sensitivity of the translated MQS were assessed by two evaluators at each hospital. For each patient, two evaluators measured the two MQS scorings daily from the time the patient was admitted to the ICU to the time the patient was discharged from the ICU. The measurement values obtained by the two assessors for each patient were blinded to those obtained by the other assessors during the testing period. The researcher obtained permission to use the version of the MQS that was developed in English and translated it into the Japanese version of the MQS. The MQS was forward-translated by two native Japanese speakers in accordance with the guidelines for the process of cross-cultural adaptation of self-report measures [14]. One of the translators was a physical therapist with several years of experience, and the other was a person with no medical knowledge or education. The two translated Japanese versions were integrated into a consensus version after the parts with vague interpretations were discussed at a consensus meeting. Backward translation was performed by two translators who were bilingual and fluent in both Japanese and English but did not have any medical knowledge. The consensus version of the forward translation was then translated back into English. The final Japanese version was completed by comparing and revising all versions of the MQS by a committee of experts, including two doctors, two nurses, and two physiotherapists (see Appendix).

Patients

The study was performed over six months, from September 1, 2022 to March 31, 2023. The study included patients who were recently admitted to the medical ICU of Nagoya Medical Center, Naha City Hospital, and Steel Memorial Yawata Hospital, Japan. All patients were on mechanical ventilation for 48 hours or longer, and physiotherapy was ordered on weekdays. The exclusion criteria were patients under 18 years of age, those with a Barthel index (BI) <70 before admission, those with neurological complications or a lack of communication skills due to pre-existing mental diseases, and those in a terminal stage. We also excluded patients who died and those who did not complete the assessment during their ICU stay, as well as those who never met the criteria for physiological stability. All patients were managed according to the early mobilization expert consensus [7].

Ethical considerations

In accordance with the Declaration of Helsinki, written informed consent was obtained from all the participants prior to the ICU discharge after explaining the purpose, expected benefits, and potential harms of the study. This study was approved by the Ethics Committee of the Gifu University of Health Sciences (approval number: 2022-04).

Assessment

The rehabilitation levels were quantified based on the ICU mobility scale (levels 1-10) [15]. To calculate the rehabilitation dose (intensity × activity time), each rehabilitation level was assigned a duration to define one unit of the MQS. The daily MQS obtained from the nursing and physiotherapy data was totaled throughout the ICU stay and then divided by the duration of the ICU stay to obtain the average daily MQS (average daily MQS = total MQS during the ICU/ICU length of stay). In addition, the mean MQS was bisected to determine the cutoff points for high-/low-dose rehabilitation as the exposure variable. The median served as the cut-off for high-/low-dose rehabilitation recruitment as a binary variable.

To investigate the agreement among the observers, the role of the evaluator was performed by two physiotherapists at each participating hospital (Nagoya Medical Center: SW, main assessor, and KK with 12 and seven years of experience working in the ICU, respectively; Naha City Hospital: DY and TT with 17 and 10 years of experience working in the ICU, respectively; Steel Memorial Yawata Hospital: KY and KG with 15 and seven years of experience working in the ICU, respectively). All the assessors were trained by the study members to apply the MQS in simulated cases and during routine care.

Data collection

The primary outcome was ADL dependence at hospital discharge. ADL dependence was defined as a BI of <70 (including hospital mortality). The following basic patient information was recorded at the time of ICU admission: age, sex, body mass index, acute physiologic assessment and chronic health evaluation (APACHE II) score, ICU admission diagnosis, ICU and hospital lengths of stay, duration of mechanical ventilation, and in-hospital mortality.

Statistical analyses

Continuous variables and categorical data were described using medians with interquartile ranges and numbers with percentages, respectively. The reliability among the assessors was verified by calculating the interclass correlation coefficient (ICC) (2.1) for the average daily MQS. ICC in the range of 0.75-0.90 was considered good, and >0.90 was excellent [16]. To examine the effectiveness, we performed a multiple logistic regression analysis with age and APACHE II scores as covariates. These factors have been shown in previous studies to be associated with adverse outcomes [17]. Logistic regression analysis was performed with ADL dependence at hospital discharge as the objective variable and the mean MQS score during all ICU admissions or the high dose of MQS as the explanatory variable. For effectiveness, we used data from the main assessor.

All analyses were performed using the JMP software (version 13.0; SAS Institute Inc., Cary, NC, USA). Statistical tests were two-sided, and p < 0.05 was considered statistically significant.

## Results

**Figure 1:**
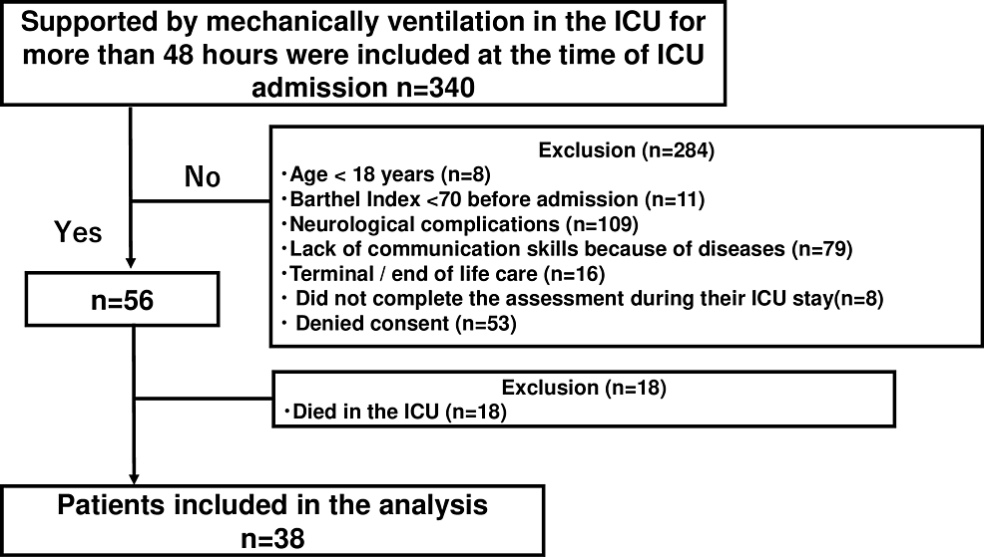
Flow chart of the patient selection process. Neurological complications include cerebral infarction, cerebral hemorrhage, acute subdual hematoma, acute epidural hematoma, traumatic subarachnoid hemorrhage, and encephalitis. ICU: intensive care unit

Of the 340 target patients, eight were aged <18 years, 109 had neurological complications, 11 had a BI <70 before admission, 79 had a lack of communication skills, 16 were terminally ill, eight did not complete the assessment during their ICU stay, 18 died in the ICU, and 53 denied consent. After 302 patients were excluded, 38 were included in the study (Figure 1). The characteristics of these patients are presented in Table 1.

**Table 1:** Characteristics and results for the mobilization quantification score (n = 38) Median (25th-75th percentile) or the number of patients (%) IQR: interquartile range; BMI: body mass index; APACHE: acute physiologic assessment and chronic health evaluation; ICU: intensive care unit; MQS: mobilization quantification score

Variable	n = 38
Age (years), median (IQR)	74.5 (63.8-82.0)
Sex (male), n (%)	21 (55%)
BMI (kg/m^2^), median (IQR)	19.0 (17.2-22.2)
ICU admission diagnosis, n (%)	
Acute respiratory failure	9 (24%)
Cardiovascular disease	5 (13%)
Gastric or colonic surgery	20 (52%)
Other diagnoses	4 (11%)
APACHE II score, median (IQR)	22.5 (17.8-26.0)
Charlson comorbidity index, median (IQR)	1 (0-3)
ICU length of stay, median (IQR)	5.6 (4.1-8.2)
Duration of mechanical ventilation, median (IQR)	3.7 (2.6-5.5)
Hospital length of stay (IQR)	26.9 (17.7-43.1)
Mean MQS score (IQR)	4.1 (2.4-7.3)
Hospital survival, n (%)	34 (89%)
Barthel index at hospital discharge (IQR)	85.0 (53.8-95.0)

Inter-assessor reliability

Six assessors, two from each hospital, were able to measure the MQS in 38 patients. The mean ICC (2.1) for the MQS mean value was 0.84 (0.78-0.91) during the ICU stay.

Effectiveness 

Logistic regression analysis using the mean MQS on admission to ICUs as an explanatory variable showed a significant association between increased MQS and decreased ADL dependence at discharge (odds ratio (OR): 0.76; confidence interval (CI): 0.61-0.96, adjusted p = 0.009) (Table 2). Logistic regression analysis using high MQS on admission to ICUs as an explanatory variable showed a significant association between increased MQS and decreased ADL dependence at discharge (OR: 0.14; CI: 0.03-0.66, adjusted p = 0.013).

**Table 2:** Multivariate logistic regression analysis of independent variables for activity of daily living independence at discharge, excluding fatal cases. MQS: mobilization quantification score; ICU: intensive care unit; OR: odds ratio; CI: confidence interval; APACHE: acute physiology and chronic health evaluation

Variable	Univariate			Model 1 adjusted for age and APACHE II		
	OR	95% CI	P value	OR	95% CI	P-value
Mean MQS during all ICU admissions	0.75	0.59-0.95	0.004	0.76	0.61-0.96	0.009
High dose of MQS (mean MQS >4.1)	0.12	0.03-0.53	0.005	0.14	0.03-0.66	0.013
Number of daily rehabilitations per person during ward	0.95	1.02-1.05	0.095	0.08	0.01-0.50	0.007

## Discussion

An examination of the reliability of an evaluation method is often based on how negligible the measurement error is when two assessors conduct the same test under the same conditions. Lamdis et al. [16] concluded that reproducibility was effective if the ICC was >0.8. Previous studies have confirmed the high inter-rater reliability of the MQS measurements. SOMS has also been reported to be good interrater reliability among excellent assessors [18]. In this study, the total and subtotal values of MQS was 0.8 or higher, demonstrating a high inter-rater reliability.

Patients admitted to the ICUs frequently develop PICS [19]. Insights into how intense PA should be targeted during the ICU stay for early recovery could help avoid increasing reliance on ADLs during hospital discharge for critically ill patients [20,21]. However, in many cases, it is difficult to perform PA assessment with a PA meter or a metabolic meter at an early stage in critically ill patients due to the effects of sedation and mechanical ventilation. In the cluster analysis of Fuest et al., the mobilization dose was determined by sessions per day, mean duration, early mobilization, and average and maximum level achieved, but it is difficult to use all these evaluations on the bedside [22]. The MQS has several benefits over other scales, including the combination of duration and intensity in a single score. This study included patients under mechanical ventilation, and high inter-rater reliability was observed even in critically ill patients. Therefore, high inter-rater reliability could be ensured, if the MQS measurement procedure is clarified and used, even in patients under mechanical ventilation.

In a previous study using the MQS, high-dose rehabilitation in the surgical ICU was an independent predictor of ADL dependence at hospital discharge [12]. Previous studies in stroke patients have similarly associated higher mobilization doses with a lower risk of losing the ability to live independently after hospital discharge [13]. In this study, we found a significant association between MQS and ADL independence, suggesting that MQS may be useful as a predictor of ADL independence. Our results highlight the need to quantify the rehabilitation activity time when investigating the effects of early rehabilitation in mechanically ventilated patients. However, in this study, MQS, which is a continuous variable, was transformed into a binary variable based on the median. For MQS above the median, a selection bias may have occurred, if all patients were included in the highly variable high-dose mobilization group.

This study has some limitations. This study lacked complete data and had a small sample size. Only 17% of the patients in the ICU were included during the study period, which is an important source of selection bias that may limit the generalizability of the findings to other ICUs. In addition, except for age and APACHE II score, factors assumed to be associated with ADL dependence at hospital discharge were not adjusted for in this study, such as the barriers to mobilization and medication. Furthermore, the reliability of the test-retest method, in which the MQS was measured twice, was not examined. In critically ill patients admitted to the ICU, the re-test method may not be highly reliable because of the effects of changes in pathology and sedation. In the future, verification of the reliability using the retest method will be required. In this study, both the assessors were physiotherapists, and it is necessary to examine whether similar results can be performed by multiple professionals, such as nurses involved in the ICU.

## Conclusions

The Japanese version of the MQS has been validated in this study. The reliability and effectiveness of the MQS have been studied. The inter-rater reliability of the MQS was high. In addition, the MQS was associated with ADL dependence at discharge, suggesting that it may be useful as a predictor of adverse physical outcomes. The MQS could ensure high inter-evaluator reproducibility if the measurement procedure was clarified and used, even under mechanical ventilation.

Further research is needed to identify and eliminate the confounding factors involved in MQS and ADL dependence.

## Figures and Tables

**Figure 2:**
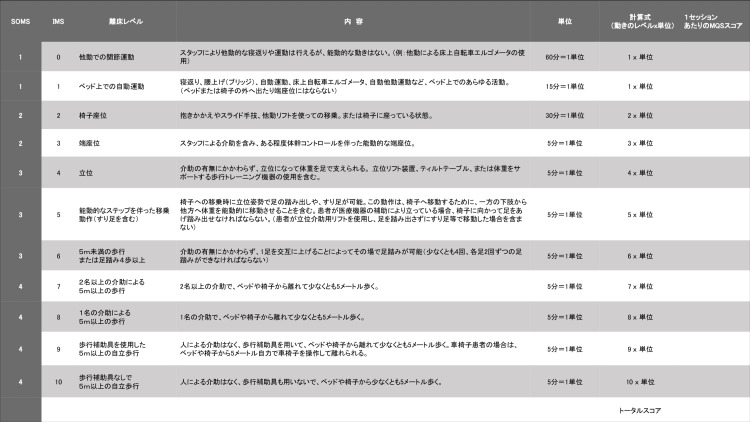
Japanese version of the mobilization quantification score SOMS: surgical intensive care unit optimal mobilization score; IMS: ICU mobility scale

## References

[1] Needham DM, Davidson J, Cohen H (2012). Improving long-term outcomes after discharge from intensive care unit: report from a stakeholders' conference. Crit Care Med.

[2] Spies CD, Krampe H, Paul N (2021). Instruments to measure outcomes of post-intensive care syndrome in outpatient care settings - Results of an expert consensus and feasibility field test. J Intensive Care Soc.

[3] Kawakami D, Fujitani S, Morimoto T (2021). Prevalence of post-intensive care syndrome among Japanese intensive care unit patients: a prospective, multicenter, observational J-PICS study. Crit Care.

[4] Watanabe S, Kotani T, Taito S (2019). Determinants of gait independence after mechanical ventilation in the intensive care unit: a Japanese multicenter retrospective exploratory cohort study. J Intensive Care.

[5] Watanabe S, Liu K, Morita Y (2021). Changes in barriers to implementing early mobilization in the intensive care unit: a single center retrospective cohort study. Nagoya J Med Sci.

[6] Watanabe S, Liu K, Nakamura K (2022). Association between early mobilization in the ICU and psychiatric symptoms after surviving a critical illness: A multi-center prospective cohort study. J Clin Med.

[7] Ad Hoc Committee for Early Rehabilitation (2017). The Japanese Society of Intensive Care Medicine: evidence based expert consensus for early rehabilitation in the intensive care unit [Article in Japanese]. Jpn J Int Care Med.

[8] Garcia-Rio F, Rojo B, Casitas R (2012). Prognostic value of the objective measurement of daily physical activity in patients with COPD. Chest.

[9] Takahashi K, Tashiro H, Tajiri R (2021). Factors associated with reduction of sedentary time following tiotropium/olodaterol therapy in treatment-naïve chronic obstructive pulmonary disease. Int J Chron Obstruct Pulmon Dis.

[10] Sallis R, Young DR, Tartof SY (2021). Physical inactivity is associated with a higher risk for severe COVID-19 outcomes: a study in 48 440 adult patients. Br J Sports Med.

[11] Parker A, Sricharoenchai T, Needham DM (2013). Early rehabilitation in the intensive care unit: preventing physical and mental health impairments. Curr Phys Med Rehabil Rep.

[12] Scheffenbichler FT, Teja B, Wongtangman K (2021). Effects of the level and duration of mobilization therapy in the surgical ICU on the loss of the ability to live independently: an international prospective cohort study. Crit Care Med.

[13] Mazwi N, Lissak I, Wongtangman K (2023). Effects of mobility dose on discharge disposition in critically-ill stroke patients. PM R.

[14] Beaton DE, Bombardier C, Guillemin F, Ferraz MB (2000). Guidelines for the process of cross-cultural adaptation of self-report measures. Spine (Phila Pa 1976).

[15] Hodgson C, Needham D, Haines K (2014). Feasibility and inter-rater reliability of the ICU Mobility Scale. Heart Lung.

[16] Koo TK, Li MY (2016). A guideline of selecting and reporting intraclass correlation coefficients for reliability research. J Chiropr Med.

[17] Kim RY, Murphy TE, Doyle M (2019). Factors associated with discharge home among medical ICU patients in an early mobilization program. Crit Care Explor.

[18] Schaller SJ, Stäuble CG, Suemasa M (2016). The German validation study of the surgical intensive care unit optimal mobility score. J Crit Care.

[19] Inoue S, Nakanishi N, Nakamura K (2022). Post-intensive care syndrome-10 years after its proposal and future directions. J Clin Med.

[20] Watanabe S, Hirasawa J, Naito Y (2023). Association between intensive care unit-acquired weakness and early nutrition and rehabilitation intensity in mechanically ventilated patients: a multicenter retrospective observational study. Cureus.

[21] Watanabe S, Hirasawa J, Naito Y (2023). Association between the early mobilization of mechanically ventilated patients and independence in activities of daily living at hospital discharge. Sci Rep.

[22] Fuest KE, Ulm B, Daum N (2023). Clustering of critically ill patients using an individualized learning approach enables dose optimization of mobilization in the ICU. Crit Care.

